# Global management of patients with knee osteoarthritis begins with quality of life assessment: a systematic review

**DOI:** 10.1186/s12891-019-2895-3

**Published:** 2019-10-27

**Authors:** Marianna Vitaloni, Angie Botto-van Bemden, Rosa Maya Sciortino Contreras, Deborah Scotton, Marco Bibas, Maritza Quintero, Jordi Monfort, Xavier Carné, Francisco de Abajo, Elizabeth Oswald, Maria R. Cabot, Marco Matucci, Patrick du Souich, Ingrid Möller, Guy Eakin, Josep Verges

**Affiliations:** 1 Osteoarthritis Foundation International OAFI, Barcelona, Spain; 2 Arthritis Foundation, Atlanta, USA; 3 De los Andes University, Merida, Venezuela; 4 Rheumatology Service, Del Mar Hospital, Barcelona, Spain; 5 Clinical Pharmacology Department, Clinic Hospital, Barcelona, Spain; 6 Clinical Pharmacology Department, Alcalá University, Madrid, Spain; 7 Faculty of Nursing, Clinic Hospital, Barcelona, Spain; 8 Rheumatology Service, University of Florence, Florence, Italy; 9 University of Montreal, Montreal, Canada; 10 Poal Institute, University of Barcelona, Barcelona, Spain

**Keywords:** Osteoarthritis, Knee, Quality of life, Individual factors, Social Determinants of Health, Psychosocial factors, Patient advocacy organizations, Patient Centred

## Abstract

**Background:**

Knee osteoarthritis (KOA) is a prevalent form of chronic joint disease associated with functional restrictions and pain. Activity limitations negatively impact social connectedness and psychological well-being, reducing the quality of life (QoL) of patients. The purpose of this review is to summarize the existing information on QoL in KOA patients and share the reported individual factors, which may influence it.

**Methods:**

We conducted a systematic review examining the literature up to JAN/2017 available at MEDLINE, EMBASE, Cochrane, and PsycINFO using KOA and QOL related keywords. Inclusion criteria were QOL compared to at least one demographic factor (e.g., age, gender), lifestyle factor (e.g., functional independence), or comorbidity factor (e.g., diabetes, obesity) and a control group. Analytical methods were not considered as part of the original design.

**Results:**

A total of 610 articles were reviewed, of which 62 met inclusion criteria. Instruments used to measure QoL included: SF-36, EQ-5D, KOOS, WHOQOL, HAS, AIMS, NHP and JKOM. All studies reported worse QoL in KOA patients when compared to a control group. When females were compared to males, females reported worse QOL. Obesity as well as lower level of physical activity were reported with lower QoL scores. Knee self-management programs delivered by healthcare professionals improved QoL in patients with KOA. Educational level and higher total mindfulness were reported to improve QoL whereas poverty, psychological distress, depression and lacking familial relationships reduce it. Surgical KOA interventions resulted in good to excellent outcomes generally; although, results varied by age, weight, and depression.

**Conclusion:**

KOA has a substantial impact on QoL. In KOA patients, QoL is also influenced by specific individual factors including gender, body weight, physical activity, mental health, and education. Importantly, education and management programs designed to support KOA patients report improved QoL. QoL data is a valuable tool providing health care professionals with a better comprehension of KOA disease to aid implementation of the most effective management plan.

## Background

Knee Osteoarthritis (KOA) is one of the primary causes of pain and disability worldwide. The pain and disability are associated with functional restrictions, morphological changes in the subchondral bone, articular cartilage degeneration and damage to the surrounding soft tissue [1–3]. In addition to the structural and functional limitations caused by KOA, pain and disability from KOA also affect social connectedness, relationships and emotional well-being; subsequently, reducing quality of life (QoL) [4]. The goal of treatment has traditionally focused on reducing pain and improving function, yet healthcare providers are increasingly realizing the importance of ensuring implementation of psychosocial support to improve the health and overall well-being of KOA patients. Assessing QoL is an imperative first step in evaluating well-being, disease progression and intervention efficacy [5–8].

Notably, measurement of QoL in KOA is increasing in research and clinical practice, but it still is not routine [9]. As far as we know, this is the first systematic review summarizing existing studies results reporting QoL in KOA patients combined with individual factors such as demographics (e.g., age, gender), lifestyle (e.g., functional independence), or comorbidities (e.g., diabetes, obesity).

The purpose of this review is to provide an international resource summarizing available studies, which have reported individual factors affecting QoL in KOA patients. Our results aim to prompt incorporation of psychosocial assessment in management strategies. Patient organization representatives designed and executed this summary to prompt routine evaluation of such.

## Methods

### Search strategy

We identified original articles using electronic searches of MEDLINE, EMBASE, Cochrane and PsycINFO databases. Literature review start date was unrestricted and end date was January 23, 2017. Searches were not limited by language. However, no eligible study was found in non-English languages. The keywords used were “knee osteoarthritis” AND “quality of life” OR “life quality” OR “wellbeing” OR “well-being” OR “short form 36” OR “knee injury and osteoarthritis outcome score” OR “koos” OR “koos-qol” OR “euroqol” OR “assessment of quality of life” OR “qualitymetric” OR “whoqol-100” OR “quality of wellbeing” OR “rosser” OR “osteoarthritis quality of life scale” OR “osteoarthritis knee and hip quality of life” OR “arthritis impact measurement scale” and all shorter forms and variations. The systematic review was conducted following Preferred Reporting Items for Systematic Reviews and Meta-Analyses guidelines [9].

### Inclusion and exclusion criteria

An example of the tag flowchart for inclusion and exclusion can be found in online Additional file 1: Figure S1. Only abstracts or articles reporting original data on QoL of KOA patients were included. Inclusion criteria were QoL associated to one or more demographic factors (e.g., age, gender), lifestyle factors (e.g., functional independence), or comorbidity factors (e.g., diabetes, obesity) and compared with a reference population, or control group. The control group was composed of individuals without KOA. There were no other restrictions on the comparison control group. There was no restriction on age, gender, language, or year of publication. Review articles, protocols for clinical trials, commentaries, editorials, proceedings summaries, or instrument development summaries were excluded from this review. Articles that described unspecified knee pain, pre-intervention anterior cruciate ligament repair, hip osteoarthritis (OA), spine OA or any study that combined KOA patients with other cohorts of patients and did not collect, analyse, or report KOA-specific data separately (e.g., a population defined as “hip or KOA” or “hip and/or KOA”) were also excluded. Three reviewers independently assessed each reference against pre-specified inclusion and exclusion criteria using a two-stage process: first, titles and abstracts, and, second, full-text articles. Any queries were resolved during a consensus meeting.

### Data extraction

A single reviewer, using a pre-piloted extraction form, obtained the data for each eligible article. Study characteristics including publication details (author and year), participant characteristics (age, sex, body mass index [BMI] and number of participants in each group), instruments, treatments applied in the intervention and control groups, and a summary of main findings were extracted from each included study for subsequent review amongst all three reviewers.

### Quality appraisal

**Fig. 1:**
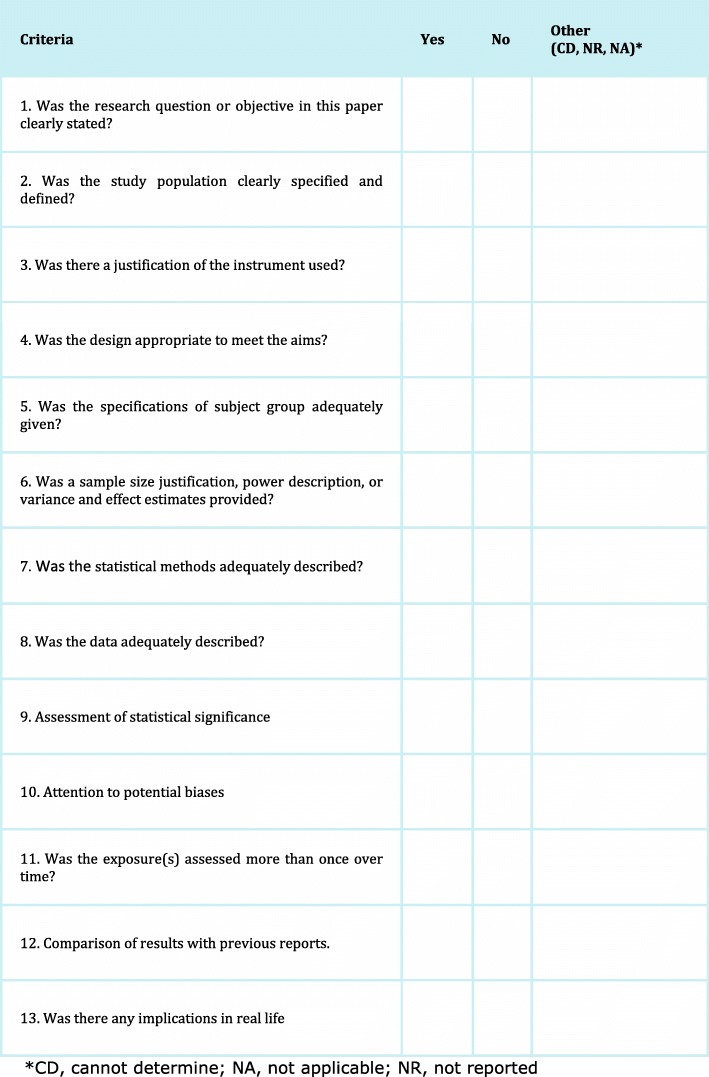
Quality Appraisal tool. The quality appraisal tool is a modify version of the quality appraisal tool recommended by Cochrane. Three independent researchers scored the 13 items

The quality assessment of each article was based on a modified version of the Cochrane quality appraisal tool [10]. The individual assessment criteria are presented in Fig. 1. Strength of consistency between raters was not scored yet individual and average quality assessment results are included in online Additional file 2. One point was allocated for each of the 13 quality appraisal criteria. The maximum score was 13 (indicating high quality), with the lowest possible score being zero. The methodological quality of each study was rated as low (0–4 points), moderate (5–9 points), or high (10–13 points).

## Results

### Literature search results

**Fig. 2:**
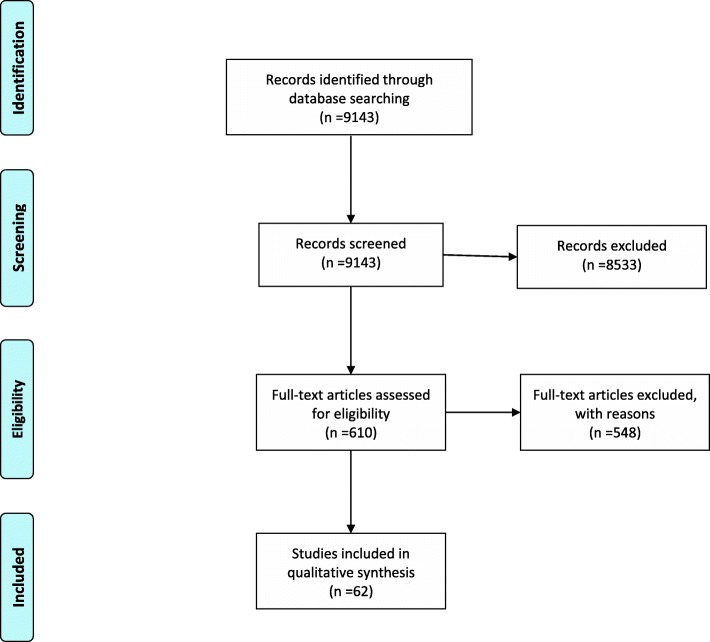
Flowchart of study selection*.* A total of 9143 articles were initially identify, 610 articles were then selected based on title and abstract screening. 62 articles were included in the final study

A total of 9143 articles were initially identified (Fig. 2); 4863 articles from EMBASE, 2792 from PubMed, 1279 from Cochrane and 209 from PsycINFO. A total of 610 articles were selected after initial title and abstract screening. After manual searches, full text review and removal of duplicates, 62 articles were included for final data extraction. Studies were labelled by first author and year of publication (Table 1). Year of publication ranged from 1995 to 2017.

**Table 1:** Overview of studies reporting QoL in patients with KOA

References	Country	Study design	QoL instrument	Total Sample Size	Control Population	KOA Patients	Mean age	Gender Distribution
Cuzdan, 2017 [11]	Turkey	Cross-Sectional	SF-36	85	25	60	65.79	Knee OA: 57 female; 3 male
								Control: 13 female; 12 male
Elbaz, 2017 [12]	Israel	Prospective observational	SF-36	93	30	63	64.2	Knee OA: 41 female; 22 male
								Control: 9 female; 21 male
Lee, 2017 [13]	USA	Cross-Sectional	SF-36	120	40	80	60.3	Knee OA: 61 female; 19 male
								Control: NA
Rundell, 2017 [14]	USA	Prospective	EQ-5D	5155	4711	368	75.3	Knee OA: 272 female; 96 male
								Control 3017 female; 1694 male
Wright, 2017 [15]	Australia	Cross-Sectional	SF-36	120	40	80	64	Knee OA: 44 female; 36 male
Araujo, 2016 [16]	Brazil	Cross-Sectional	SF-12	93		93	60	Knee OA: 69 female; 24 male Control: 24 female; 16 male
Bokaeian, 2016 [17]	Iran	Randomized clinical trial	WOMAC	28		28	52.9	Knee OA: 25 female; 2 male
Cho, 2016 [18]	Republic of Korea	Prospective cohort study	SF-36	681			71.9	Knee OA: 383 female; 298 male
Kaban, 2016 [19]	Turkey	Cross-Sectional	SF-36	63	21	42	56.86	All female
Gomes-Neto, 2016 [20]	Brazil	Cross-Sectional	SF-36	35		35	66.57	Knee OA: 29 female; 6 male
Khatib, 2016 [21]	Australia	Cross-Sectional	Tot. EQ (adjusted from EQ-5D-5L)	2809		2809	68	Knee OA: 1740 female; 1069 male
Kiadaliri, 2016 [22]	Sweden	Population based cohort study	EQ-5D	1501	744	402	71.5	Group 1 (reference group having neither knee pain nor radiographic or clinically-defined knee OA) 469 female; 275 male
								Group 2 (knee pain with-out OA) 119 female; 50 male
								Group 3 (kne epain with OA) 256 female; 146 male
Kiadaliri, 2016 [23]^a^	Sweden	Retrospective	EQ-5D	First stage 7402; Second stage 1527		The number of people diagnosis with knee OA is not specified	69.4	First stage 4604 female; 2798 male
								Second stage 977 female; 550 male
Oishi, 2016 [24]	Japan	Cross-Sectional	KOOS	963		397	54.33	Total: 595 female; 368 male
								Knee OA: 299 female; 98 male
Sarumathy, 2016 [25]	India	Prospective study	SF-36	74		74	51.7	Knee OA: 55 female; 19 male
Cavalcante, 2015 [26]	Brazil	Cross-Sectional	WHOQOL	90	40	50	67	All female
Fang, 2015 [27]	Taiwan	Population based study	SF-12	901	441	460	74.04	Total: 492 female; 409 male
								Knee OA: 232 female; 209 male
Ferreira, 2015 [28]	Brazil	Cross-Sectional	SF-36	75	35	40	68.36	All female
Kawano, 2015 [29]	Brazil	Cross-Sectional	SF-36	93		93	61.2	Knee OA: 69 females; 24 male
Kim, 2015 [30]	Korea	Cross-Sectional	EQ-5D	2165		2165	67.73	Knee OA: 1458 female; 707 male
Lee, 2015 [31]	South Korea	Cross-Sectional	EQ-5D	7977		7977	61.5	Knee OA: 5448 female; 4064 male
Pang, 2015 [32]	China	Cross-Sectional	SF-36	466		466	56.56	Knee OA: 382 female; 84 male
Rakel, 2015 [33]	USA	Cross-Sectional	SF-36	100	25	75	56	Knee OA: 46 female; 29 male
								Control: 15 female; 10 male
Reid, 2015 [34]	USA	Randomized controlled trial	SF-36	190		190	60.2	Knee OA: 132 female; 58 male
Tsonga, 2015 [35]	Greece	Longitudinal	SF-36	68		68	73	Knee OA: 57 female; 11 male
Visser, 2015 [36]	Netherlands	Cross-Sectional	SF-36	1262	1060	205	56	Total: 707 female; 578 male
								Knee OA: 125 female; 80 male
								Control: 583 female; 477 male
Alburquerque-garcía, 2015 [37]	Spain	Cross-Sectional	SF-36	36	18	18	85	All female
Alkan, 2014 [38]	Turkey	Cross-Sectional	SF-36	152	40	112	60	Knee OA: 85 female; 27 male
								Control: 30 female 10 male
Forkel, 2014 [39]	Germany	Cross-Sectional	KOOS	22		22	47	Knee OA: 17 female; 6 male
Jahnke, 2014 [40]	Germany	Cross-Sectional	HAS	159		159	63.5	Knee OA: 75 female; 84 male
Marks, 2014 [41]	USA	Cross-Sectional	AIMS	21		21	70.8	All female
Pérez-Prieto, 2014 [42]	Spain	Prospective cohort study	SF-36	716		716	72	Knee OA: 421 female; 295 male
Reis, 2014 [43]	Brazil	Cross-Sectional	WHOQOL	12		12	67.25	All female
Alentorn, 2013 [44]	Spain	Cross-Sectional	SF-36	391		391	70.7	Knee OA: 303 female; 89 male
Clement, 2013 [45]	UK	Cross-Sectional	SF-12	996		996	70.32	Knee OA: 545 female; 421 male
Vulcano, 2013 [46]	USA	Prospective cohort study	SF-36	4732		4732	66.88	Knee OA: 2881 female; 1851 male
Williams, 2013 [47]	UK	Cross-Sectional	EQ-5D	2456		2456	71.4	Knee OA: 1494 female; 962 male
Coleman, 2012 [48]	Australia	Cross-Sectional	SF-36	146		146	65	Knee OA: 109 female; 37 male
Gonçalves, 2012 [49]	Portugal	Cross-Sectional	SF-36	136		136	67.2	Knee OA: 94 female; 42 male
Lim, 2012 [50]	Philippine	Cross-Sectional	WOMAC	90			70.14	Knee OA: 68 female; 22 male
Elbaz, 2011 [51]	Israel	Cross-Sectional	SF-36	1487		1487	61.9	Knee OA: 950 female; 537 male
Gonçalves, 2011 [52]	Portugal	Cross-Sectional	KOOS	377		377	67.8	Knee OA: 282 females; 95 males
Norimatsu, 2011 [53]	Japan	Prospective population-based cohort study	Japanese Knee Osteoarthritis Measure (JKOM)	333		333	64.2	All female
Ozcakir, 2011 [54]	Turkey	Cross-Sectional	NHP	100		100	59.5	Knee OA: 83 female; 17 male
Paker, 2011 [55]	Turkey	Cross-Sectional	SF-36	75		75	66.1	All female
Foroughi, 2010 [56]	Australia	Cross-Sectional	SF-36	17		17	66	All female
Jenkins, 2010 [57]	USA	Cross-Sectional	QLI-A	75		75	69	Knee OA: 57 female; 18 male
Kim, 2010 [58]	Korea	Prospective cohort study	WOMAC	504		504	70.2	Knee OA: 274 female; 230 male
Muraki, 2010 [59]	Japan	Cross-Sectional	SF-8	2126		2126	68.9	Knee OA: 1359 female; 767 male
Watanabe, 2010 [60]	Japan	Cross-Sectional	Japanese Knee Osteoarthritis Measure (JKOM)	18		18	67	All female
Yildiz, 2010 [61]	Turkey	Cross-Sectional	NHP	140		140	59.39	Knee OA: 104 females; 36 males
Debi, 2009 [62]	Israel	Cross-Sectional	SF-36	134			66.95	Knee OA: 85 females; 49 males
Imamura, 2008 [63]	Brazil	Cross-Sectional	SF-36	84	22	62	71.1	All female
							Control 68.95	
Liikavainio, 2008 [64]	Finland	Cross-Sectional	RAND-36	107	53	54	59	All male
							Control 59.24	
Wang, 2008 [65]	Germany	Cross-Sectional	SF-36	1009		1009	48.5	Knee OA: 620 female; 389 male
Nunez, 2007 [66]	Spain	Cross-Sectional	SF-36	100		100	71.2	Knee OA: 71 female; 29 male
Salaffi, 2005 [67]	Italy	Cross-Sectional	SF-36	264		KneeOA 108	65.7	Knee OA: 64 female; 44 male
						Knee OA + Hip OA 51		Knee OA + Hip OA: 32 female; 19 male
Chacón, 2004 [68]	Venezuela	Cross-Sectional	AIMS	126		126	64	Knee OA: 106 female; 20 male
Lam, 2000 [69]	China	Cross-Sectional case–control study	COOP/WONCA	760		760	57.6	Knee OA: 538 female; 222 male
de Leeuw, 1998 [70]	UK	Prospective trial	Rosser Index Matrix	101		101	71.5	Knee OA: 62 female; 39 male
Donnell, 1998 [71]	France	Cross-Sectional	Rosser Index Matrix	221		221	No specified	Knee OA: 174 female; 47 male
Ries, 1995 [72]	USA	Cross-Sectional	AIMS	47		47	69.2	Knee OA: 44 female; 3 male

SF-36/SF-12/SF-8 (n=34); EQ-5D (n=6); KOOS (n=3); AIMS(n=3); WOMAC(n=3); Rossser Index matrix (n=2); NHP (n=2);JKOM(n=2); WHOQOL(n=2);COOP/WONCA (n=1); HAS(n=1); RAND-36(n=1)

^a^Not possible to add this article in gender calculation

### Characteristics of included studies

Most of the studies were conducted in Europe (*n* = 20), followed by the North, Central and South America (*n* = 16), Asia (*n* = 12) and other (*n* = 14). The 74% (*n* = 46) of studies were cross-sectional in design, followed by other designs comprising 6% (*n* = 4) prospective cohorts, 5% (*n* = 3) prospective, 3% (*n* = 2) randomized controls trials, (*n* = 1) population based cohorts, (*n* = 1) retrospective, etc. The main instruments used to assess QoL were the SF-36 (*n* = 31) followed by EQ-5D (*n* = 6) (Table 1). The results from the quality appraisal tool had all articles scoring moderate to high quality scores; no study scored lower than 8 points. Four (6%) of the articles [25, 28, 54, 60] were classified as moderate quality, with the remaining 58 (94%) articles classified as having high quality [11–24, 26, 27, 29–53, 55–59, 61–72].

### Characteristics of participants

The total KOA population was 24,706 of which 93.4% (*n* = 23,079) were female [11–72]. 11 articles included only females [19, 26, 28, 37, 41, 43, 53, 55, 56, 60, 63] and one article included only males [64]. When the male and female only articles were removed, the total KOA population still comprised 93.8% female. The mean number of KOA patients per study was 560, with sample size varying between 12 [43] and 7977 [31] and the median number was 101. The mean age across all studies was 65 years (range 47 to 85) and 68.2 (range 56.9 to 71.1) years for those studies including only females [19, 26, 28, 37, 41, 43, 53, 55, 56, 60, 63] and 50 (range 50 to 69) for the one study enrolling only males (Table 1) [64].

### QoL in patients with KOA versus reference populations

Studies reported worse QoL for the KOA group, regardless of measurement used to assess QoL [12, 14, 22, 28, 31, 33, 38, 46, 53, 60, 61, 63, 65–67, 69]. Lower QoL scores were mostly reported with increasing age [25, 26, 32, 35, 44, 53] yet Jenkins reported higher QoL in older patients [57]. When QoL scores were compared based on gender, females with KOA reported worse QoL scores and psychosocial variables [49, 58, 66]. In online Additional file 3: Table S1 additional results are presented for studies comparing QoL of KOA patients with a reference population by instrument.

### QoL and healthy weight

Weight was reported as effecting QoL in KOA patients [11, 20, 46, 67, 70]. Vulcano, Elbaz and Visser reported a higher BMI aggravated KOA patient symptoms [11, 46, 51]. Gomez-Neto found a negative impact on functional capacity in obese KOA patients; however, found no difference reported in QoL [20]. de Leeuw, reported that the median pre-operative QoL scores for obese patients were significantly lower than for non-obese [70].

### QoL and Physical Activity

Calvacante et al. found that KOA in older women can promote a decline in time spent performing physical activity and functional fitness with decline in QoL and an increase in sitting time [26]. Wantanabe, reported reduced physical activity resulted in worse QoL and also reported too much physical activity may exacerbate the development of KOA [60]. Strength training (ST) had a positive effect in KOA patients [17]. Reid found that muscle power is an independent determinant of QoL in KOA [34].

### QoL and educational programs

Coleman evaluated a self-management educational program delivered by health care professionals, reporting an improved QoL in KOA patients assessed at 8 weeks and 6 months based on WOMAC and SF-36 measures [48].

### QoL and psychosocial factors

Higher educational level and higher total mindfulness scores were reported to improve patients’ perception of QoL [13, 23, 28]. Alkan et al. reported that approximately 70% of the study participants had low-middle education, resulting in poor QoL in this group [38]. Another study reported lower Health-Related Quality of Life (HRQL) scores for KOA females with chronic physical or mental health conditions [62]. Poverty also worsened QoL in KOA patients [45]. Psychological distress and depression worsened QoL [23, 28, 42, 54]. Patients lacking familial relationships reported worse QoL [50].

### QoL and surgical interventions

The majority of QoL findings with surgical intervention were reported as dependents of demographic variables, which showed an effect on QoL after surgical outcomes. Williams et al. [47], for example, reported that patient satisfaction was lower among patients younger than 55 years of age compared to older patients. Vulcano, reported a higher BMI was associated with worse outcomes [46]. Perez-Prieto, reported patients with depression had less improvement that non-depressed patients after surgical intervention, reporting lower QoL scores [42]. Lower socioeconomic groups undergoing surgical intervention also reported worse QoL [45]. Patients participating in sports pre- and post- surgical intervention reported higher QoL scores [40].

## Discussion

This systematic review aimed to broaden the amount of QoL data available for summarizing, using less stringent search criteria; for example, inclusion of articles irrespective of QoL being the primary endpoint. This review included surgical and non-surgical data including QoL measurement.

The broad search strategy identified 62 articles as reporting information on QoL associated to one other factor (demographic, lifestyle, or comorbidity) between KOA patients and control patients. Article details are presented in Table 1. The quality appraisal tool revealed all studies as moderate to high quality yet caution should be taken in the interpretation of findings, as this tool may not discern scientific & analytic rigour assessing QoL but scores prioritizing criteria description. For example, describing sample size, potential biases, etc. would be sufficient to permit a high score even with low analytic rigour.

It is well known that patient characteristics influence QoL. In this summary, increasing age showed worse QoL in most studies; however, in an older age group of KOA patients awaiting total knee replacement better QoL scores were reported [57]. This was a convenience sample with predominantly married, white females in a higher socioeconomic class which may have biased the results for higher QoL scores. Notably, prior reports have demonstrated that younger populations have higher expectations in terms of QoL as they expect to perform better on many activities of daily living, work and recreation [73]. It is also known that gender effects QoL in KOA patients, as reported here in a prospective aging cohort study by Kim et al. [58] the percentage of males and females were similar. Females reported worse QoL than males with KOA and females had significantly higher risk for belonging to the worst quartile for all WOMAC subscales compared to males regardless of KOA presence after adjustment of age, BMI and OA severity. Goncalves reported similar results in most SF-36 subscales [49]. Most studies in this summary comprised an above average percentage of females beyond KOA population norms which may have revealed bias toward lower QoL scores if a risk assessment for bias had been performed.

Similar to recent reports, lifestyle factors and common social determinants of health, such as unhealthy weight, low physical activity, low socioeconomic and education levels were found to have a negative impact on QoL in KOA patients [20, 38, 45, 60]. Understandably quantitative data is lacking in this summary, yet qualitative summaries and recent quantitative reports emphasize the importance of assessing these factors and implementing a whole person approach to healthcare. Health promotion and self-management strategies addressing unhealthy weight and low levels of physical activity may improve QoL. Obesity data highlights a gap and opportunity to improve KOA QoL scores by incorporating dietary guidelines and nutritional education [20, 74]. Programs where patients participate in education and supervised exercise delivered by trained physiotherapists can improve physical activity and QoL. Moreover, exercise therapy may postpone total joint replacement in patients with OA [75]. Low educational level increases the chance of having OA, and results in decreases in patients’ self-perception of QoL [76]. This relationship between education and the socioeconomic level is well recognized as individuals with lower education levels are generally relegated to manual or repetitive occupational activities increasing their risk of OA.

QoL is a powerful indicator to consider when implementing and evaluating OA management programs. QoL is best monitored and reassessed in the short and long term to ensure effectiveness of interventions. Available data on QoL interventions can be customized considering individual characteristics to improve the factors open to modulation such as weight, physical activity, emotional health and social connectedness. Management strategies may be optimized by adapting to patient-specific needs with a multimodal personalized OA management plan grounded on evidence-based therapies for whole person care.

### Limitations

There are limitations to acknowledge and use as opportunities to improve quality of future OA research and reporting to have a more meaningful impact for OA patients. The database search did not include SPORTDiscus or Cumulative Index to Nursing and Allied Health Literature (CINAHL) which may have provided additional QoL study data, particularly assessing QoL related to healthy weight, physical activity, educational and psychosocial factors. The search was limited to 2017 and additional studies may have been published thereafter reporting on QoL in KOA patients. Caution should be used when interpreting these findings, as studies were included which may not have been scientifically rigorous upon review, yet methodological reporting sufficed to meet the data extraction criteria. For example, a moderate to high score could be obtained for providing an adequate description of most criteria even if the sample size and statistical methods weren’t methodologically and analytically rigorous. Articles were included whether power analysis was performed on QoL as the primary outcome or not; thus, sample size may not have been sufficient to find differences when not powered for QoL as the primary outcome. Risk of bias was not calculated. The methodological heterogeneity of the studies limited unbiased comparisons and quantitative syntheses was not permissible.

## Conclusion

KOA studies routinely include pain and function scores yet haven’t routinely included psychosocial variables assessing QoL, which also influences how patients feel, function, and survive [77]. Unfortunately, there is no consensus on the core domains of QoL. Ensuring a standard QoL assessment is implemented, as routine care globally is imperative for healthcare professionals to gain a better understanding of OA disease whilst ensuring most optimal management.

This study was coordinated by patient organizations and previously promoted at the 2019 OARSI Annual Meeting advocating for routine assessment and on-going evaluation of QoL with implementation of a single agreed upon QoL measure [78]. Future KOA QoL research should combine efforts globally and focus on consistent quantitative and qualitative measures for more meaningful impact and interpretation.

## Supplementary information

**Additional file 1: Figure S1.** Flowchart used in the selection of the articles included in the study. The flowchart shows the sequence of criteria followed for the selection of the articles included in the study.

**Additional file 2.** Quality appraisal report results obtained after the evaluation of articles quality based on the Cochrane modified instrument rated by three independent reviewers and the mean value of the three scores.

**Additional file 3: Table S1.** Quality of life of KOA patients vs. reference population by instrument used. Table comparing the variable values in KOA patient’s population with reference populations and statistical significance analysis. The data are organized by article.

## Data Availability

The source of data has been included in the manuscript.

## Abbreviations

CINAHL: Cumulative Index to Nursing and Allied Health Literature
BMI: Body mass index
HAS: Heidelberg Sports Activity Score
HRQL: Health-Related Quality of Life
KOA: Knee Osteoarthritis
OA: Osteoarthritis
QoL: Quality of life
ST: Strengthening training
TKR: Total knee replacement
UKA: Uncompartmentalized knee arthroplasty
