# Supplementary material for: Global management of patients with knee osteoarthritis begins with quality of life assessment: a systematic review
Source: BMC Musculoskelet Disord. 2019 Oct 27;20:493. doi: 10.1186/s12891-019-2895-3 (PMC6815415; doi:10.1186/s12891-019-2895-3)
Supplement: Supplementary file 1 — Additional file 1: Figure S1. Flowchart used in the selection of the articles included in the study. The flowchart shows the sequence of criteria followed for the selection of the articles included in the study. [file 12891_2019_2895_MOESM1_ESM.pptx]

## Slide 1
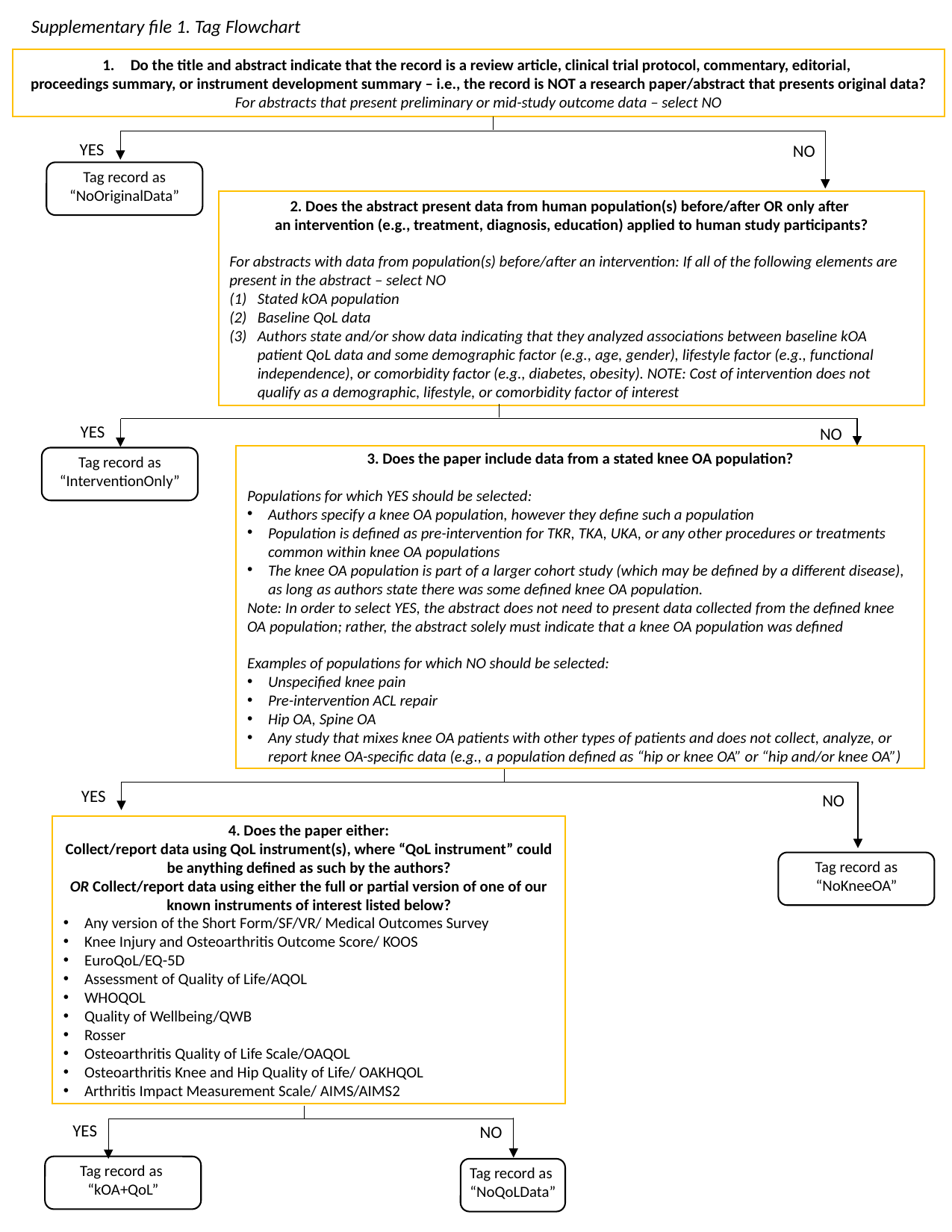

Supplementary file 1. Tag Flowchart
Do the title and abstract indicate that the record is a review article, clinical trial protocol, commentary, editorial,
proceedings summary, or instrument development summary – i.e., the record is NOT a research paper/abstract that presents original data?
For abstracts that present preliminary or mid-study outcome data – select NO
YES
NO
Tag record as “NoOriginalData”
2. Does the abstract present data from human population(s) before/after OR only after
an intervention (e.g., treatment, diagnosis, education) applied to human study participants?
For abstracts with data from population(s) before/after an intervention: If all of the following elements are present in the abstract – select NO
Stated kOA population
Baseline QoL data
Authors state and/or show data indicating that they analyzed associations between baseline kOA patient QoL data and some demographic factor (e.g., age, gender), lifestyle factor (e.g., functional independence), or comorbidity factor (e.g., diabetes, obesity). NOTE: Cost of intervention does not qualify as a demographic, lifestyle, or comorbidity factor of interest
YES
NO
3. Does the paper include data from a stated knee OA population?
Populations for which YES should be selected:
Authors specify a knee OA population, however they define such a population
Population is defined as pre-intervention for TKR, TKA, UKA, or any other procedures or treatments common within knee OA populations
The knee OA population is part of a larger cohort study (which may be defined by a different disease), as long as authors state there was some defined knee OA population.
Note: In order to select YES, the abstract does not need to present data collected from the defined knee OA population; rather, the abstract solely must indicate that a knee OA population was defined
Examples of populations for which NO should be selected:
Unspecified knee pain
Pre-intervention ACL repair
Hip OA, Spine OA
Any study that mixes knee OA patients with other types of patients and does not collect, analyze, or report knee OA-specific data (e.g., a population defined as “hip or knee OA” or “hip and/or knee OA”)
Tag record as “InterventionOnly”
YES
NO
4. Does the paper either:
Collect/report data using QoL instrument(s), where “QoL instrument” could be anything defined as such by the authors?
OR Collect/report data using either the full or partial version of one of our known instruments of interest listed below?
Any version of the Short Form/SF/VR/ Medical Outcomes Survey
Knee Injury and Osteoarthritis Outcome Score/ KOOS
EuroQoL/EQ-5D
Assessment of Quality of Life/AQOL
WHOQOL
Quality of Wellbeing/QWB
Rosser
Osteoarthritis Quality of Life Scale/OAQOL
Osteoarthritis Knee and Hip Quality of Life/ OAKHQOL
Arthritis Impact Measurement Scale/ AIMS/AIMS2
Tag record as “NoKneeOA”
YES
NO
Tag record as
“kOA+QoL”
Tag record as
“NoQoLData”
