# Supplementary material for: Global management of patients with knee osteoarthritis begins with quality of life assessment: a systematic review
Source: BMC Musculoskelet Disord. 2019 Oct 27;20:493. doi: 10.1186/s12891-019-2895-3 (PMC6815415; doi:10.1186/s12891-019-2895-3)
Supplement: Supplementary file 3 — Additional file 3: Table S1. Quality of life of KOA patients vs. reference population by instrument used. Table comparing the variable values in KOA patient’s population with reference populations and statistical significance analysis. The data are organized by article. [file 12891_2019_2895_MOESM3_ESM.docx]

**Supplementary file 3. Table 1Supp. Quality of life of KOA patients vs. reference population.** Results of studies comparing QoL of Koa patients with reference population by instrument used.

| **Author and Year** | **Instrument** | **Results** |
| --- | --- | --- |
| Wright, 2017^15^ | SF-36 | **Worse:** physical health sub-scale of the SF-36 p=0.01.  **No Difference:** mental health sub-scale (p=0.513) |
| Kaban, 2016^19^ | SF-36 | **Worse:** negative correlation between SF-36 scores and cartilage clarity (left knee, r=−0.263, p=0.093; right knee, r=−0.312, p=0.044). |
| Elbaz, 2017^12^ | SF-36 | **Worse:** KOA50.0±16.3, Control:83.3±10.5 p<0.001 |
| Ferreira, 2015^28^ | SF-36 | **Worse:** patients with OA had a significantly lower score in all domains examined by SF-36.  **Physical functioning:**  OA:44.25(25.53), Non-OA:78.53(21.20) p<0.001.  **Role limitations due to physical health:**  OA:38.75(41.97), Non-OA:80.88(30.81) p<0.001.  **Pain:**  OA:38.45(26.50), Non-OA:75.76(20.41) p<0.001.  **General health status:**  OA:56.53(28.09), Non-OA:75.38(20.87) p<0.001.  **Vitality:**  OA:52.13(27.31), Non-OA:66.91(20.82) p<0.010.  **Social role functioning:**  OA:58.75(29.03), Non-OA:78.68(19.35) p<0.001.  **Role limitations due to emotional problems:**  OA:52.50(43.29), Non-OA:80.39(32.94),p< 0.002.  **Mental health:**  OA:57.50(25.33), Non-OA:77.29(15.72), p< 0.001. |
| Visser, 2015^36^ | SF-36 | **Worse:**  **Physical component summary** score: KOA:46.969.5, No KOA:54.267.9.  **No difference:**  **Mental component summary** score: KOA:51.269.9, No KOA:51.568.5. |
| Rakel, 2015^33^ | SF-36 | **Worse:**  **Physical functioning** Mean (SEM) KOA 38.58 (2.14) Controls 90.8 (2.58) < 0.001 Median (range) 40 (5-75) 100 (55-100)  **Role—physical**  Mean (SEM) KOA 30.33 (4.12) Controls 87 (5.80) < 0.001 Median (range) 25 (0-100) 100 (0-100)  **Bodily pain**  Mean (SEM) KOA 45.97 (2.28) Controls 89.7 (2.14) < 0.001 Median (range) 45 (0-100) 90 (67.5-100)  **General health**  Mean (SEM) KOA64.59 (2.34) Controls 82.4 (2.90) < 0.001 Median (range) 70 (0-100) 85 (55-100)  **Physical health**  Mean (SEM) KOA 44.93 (2.10) Controls 87.5 (2.32) < 0.001 Median (range) 44 (12-88) 90 (54-100)  **Vitality**  Mean (SEM) KOA 49.2 (2.50) Controls 70.6 (3.53) < 0.001 Median (range) 50 (0-100) 75 (20-100)  **Social functioning**  Mean (SEM) KOA 73.3 (2.57) Controls 94 (2.51) < 0.001 Median (range) 75 (25-100) 100 (50-100)  **Role—emotional**  Mean (SEM) KOA 66.67 (4.74) Controls 93.3 (3.85) 0.003 Median (range) 100 (0-100) 100 (33.33-100)  **Emotional well-being**  Mean (SEM) KOA 75.79 (1.82) Controls 84.5 (2.25) 0.014 Median (range) 76 (28-100) 88 (60-100)  **Mental health**  Mean (SEM) KOA 66.25 (2.27) Controls 85.60 (2.30) < 0.001 Median (range) 69.13 (17.63-100) 90.5 (49.58-100) |
| Cho, 2016^18^ | SF-36 | **No Difference**  **Physical component summary**:  No OA 59.8 (12.4) arthritis 57.1 (13.8) (NS) **Mental component summary**:  No OA 54.1 (9.3) Isolated PF 52.2 (12.1) (NS) |
| Alburquerque-García, 2014^37^ | SF-36 | **Worse:** SF-36 physical function a KOA 12.5 ± 12.5 33.1 ± 16.1 Healthy control t = −4.262; P<0.001 **No difference:**  SF-36 physical role KOA 56.9 ± 40.0 73.6 ± 33.7 Healthy control t = −1.351; P = .186 SF-36 bodily pain KOA 56.3 ± 22.7 61.5 ± 26.5 Healthy control t = −0.640; P = .527 SF-36 social function KOA 79.9 ± 23.1 79.2 ± 18.6 Healthy control t = 0.099; P = .922 SF-36 mental health KOA 55.8 ± 20.9 52.0 ± 22.5 Healthy control t = 0.521; P = .606 SF-36 emotional role KOA 68.5 ± 37.0 57.4 ± 40.9 Healthy control t = 0.854; P = .399 SF-36 vitality KOA 54.4 ± 17.9 53.6 ± 24.7 Healthy control t = 0.116; P = .909 SF-36 general health KOA 60.6 ± 16.1 59.2 ± 21.8 Healthy control t = 0.217; P = .830 |
| Alkan, 2014^38^ | SF-36 | **Worse:**  **Physical function:**  KOA 50 (0–100) Control 77.50 (50–100) P<0.000 **Role physical**:  KOA 25 (0–100) Control 100 (0–100) P<0.000 **Bodily pain**:  KOA 41 (0–100) Control 72 (40–100) P<0.000* **Vitality**:  KOA 25 (0–90) Control 55 (20–75) P<0.000 **Social function**:  KOA 50 (0–100) Control 87.50 (50–100) P<0.000 **Mental health**:  KOA 52 (4–92) Control 62 (36–100) P<0.001  **No difference:**  **General health**:  KOA 57 (0–97) Control 59.50 (25–82) (NS) **Role emotional**:  KOA 100 (0–100) Control 100 (0–100) (NS) |
| Vulcano, 2013^46^ | SF-36 | **Worse:**  **Physical Component:**  Normal Weight:36.1±8.3, Overweight: 35.1±8.1, Obesity class I:32.9±7.9, Obesity class II:31.1±7.5 Obesity class III:29.2±7.1, p<0.0001. **Mental Component:**  Normal Weight:51.6±11.6, overweight:52.2±11.5, Obesity class I:50.5±12.4, Obesity class II: 49.7±12.4, Obesity class III:48.8±13.0, p<0.0001. |
| Foroughi, 2010^56^ | SF-36 | **Worse:**  **Physical functioning:** OA: 80 (55) , Control: 95 (55) P<0.001 **Bodily pain:** OA: 59 ± 17 , Control: 85 ± 19 P<0.003 **No difference:**  **Role limitations due to physical difﬁculties:** OA: 87 (50) , Control: 100 (75) (NS) **General health:** OA: 70 (60) , Control: 70 (30) (NS) **Vitality:** OA: 63 ± 16 , Control: 58 ± 18 (NS) **Social functioning:** OA: 100 (87) , Control: 100 (62) (NS) **Role limitation due to emotional difﬁculties:** OA: 100 (50) , Control: 100 (58) (NS) **Mental health:** OA: 85 (50) , Control: 85 (55) (NS) |
| Wang, 2008^65^ | SF-36 | **Worse:** p<0.01  OA:  **Physical functioning:** 62.7  **Role-physical:** 46.7  **Bodily pain:** 48.8,  **General Health**:53.0*  **Vitality**:57.7  **Social functioning:** 80.2,  **Role limitation due to emotional difﬁculties**:89.6*,  **Mental Health**:72.6.  Reference population:  **Physical functioning:** 82.8  **Role-physical:** 69.1,  **Bodily pain:** 68.6,  **General Health**:64.0,  **Vitality**:54.1,  **Social functioning:** 77.7  **Role limitation due to emotional difﬁculties** 72.6,  **Mental Health**:67.0. |
| Imamura, 2008^63^ | SF-36 | **Worse:** SF-36 score, median (range) **Physical functioning,** KOA: 15.0 (0–65) Control: 92.5 (40–100) p<0.001 **Role-physical**, KOA: 0 (0–75), Control: 100 (0–100) p<0.001 **Bodily pain:** KOA: 22.0 (0–61), Control: 84.0 (61–100) p<0.001 **Vitality:** KOA: 70.0 (0–95), Control: 87.5 (35–130) p<0.001 **Social functioning:** KOA: 35.51 (12.5–87.5), Control: 100 (37.5–100) p< 0.001  **No difference:**  **General health:** KOA: 71.5 (20–95), Control: 82.0 (5–100) (NS):0.006 **Role-emotional:** KOA: 100 (0–100), Control: 100 (0–100) (NS):0.460 **Mental health:** KOA: 72.0 (36–92), Control: 92 (24–100) (NS):0.0002.  Lower PPT values were correlated with higher pain intensity, higher disability scores, and with poorer quality of life values, except for the role-emotional and general health status. |
| Nunez, 2007^66^ | SF-36 | **Worse:** HRQL measured by SF-36 than the reference population, mainly in physical function, physical role and bodily pain dimensions (P < 0.05). |
| Salaffi, 2005^67^ | SF-36 | **Worse:**  **Physical function** KOA: 48.1, Control group: 79.2.  **Role limitations (physical)** KOA: 33.0, Control group: 79.2.  **Bodily pain** KOA: 31.1, Control group: 76.3.  **Energy/vitality** KOA: 51.4, Control group: 74.1.  **Role limitation (emotional)** KOA: 44.9, Control group: 79.2.  **Mental health** KOA: 60.0, Control group: 80.4.  **Social function** KOA: 60.4, Control group: 84.0.  **General health perceptions** KOA: 51.9, Control group: 76.1.  **SF-36**  **Physical Component Summary** (mean=50; SD=10) KOA: 34.9, Control group: 51.9. SF-36  **Mental Component Summary** (mean=50; SD=10) KOA: 40.9 Control group: 53.6 P<0.0001. |
| Rundell, 2017^14^ | EQ-5D | **Worse:** Non-OA0.76±0.17, OA:0.72±0.18 |
| Lee, 2017^31^ | EQ-5D | **Worse:**  Men: KOA: 0.91 (0.89–0.92), Normal: 0.94 (0.94–0.95) < 0.001,  Women: KOA:0.84 (0.83–0.85), Normal: 0.91 (0.90–0.92) < 0.001.  Symptomatic radiographic knee osteoarthritis:  EQ-5D index:  Men: KOA: 0.78 (0.75–0.81), Normal 0.94 (0.94–0.95) < 0.001  Women: KOA: 0.74 (0.72–0.76), Normal: 0.91 (0.91–0.92) < 0.001 |
| Kiadaliri, 2016^22^ | EQ-5D-3L | **Worse:**  **Mobility**:  Reference group: 11.5 (8.9 to 14.8), Knee pain with KOA: 52.2 (44.8 to 59.5), Radiographic KOA without knee pain: 34.7 (25.4 to 45.5) P < 0.05 **Self-care:** Reference group: 0.2 (0.1 to 0.5) , Knee pain with KOA:7.3 (3.5 to 14.5), Radiographic KOA without knee pain: 3.6 (1.0 to 12.3). P < 0.05 **Usual activities:** Reference group: 5.6 (3.9 to 8.0), Knee pain with KOA: 24.8 (20.1 to 30.3), Radiographic KOA without knee pain: 10.0 (5.1 to 18.7). P < 0.05 **Pain/discomfort:** Reference group: 35.9 (31.8 to 40.3), Knee pain with KOA: 96.7 (92.1 to 98.7), Radiographic KOA without knee pain:70.4 (60.1 to 78.9). P < 0.05 **Anxiety/depression:** Reference group: 17.5 (14.3 to 21.1), Knee pain with KOA:32.2 (25.9 to 39.4), Radiographic KOA without knee pain: 21.4 (13.9 to 31.4). P < 0.05 |
| Watanabe, 2010^60^ | Japanese Knee Osteoarthritis Measure (JKOM) | **Worse:** Net energy expenditure (r= –0.65, p=0.04) and (b) step counts (r= –0.62, p=0.02) are negatively correlated to Japanese Knee Osteoarthritis Measure (JKOM) scores in patients with knee osteoarthritis, but not in controls (r= –0.31, p=0.26 and r= –0.39, p=0.15, respectively). |
| Norimatsu, 2011^53^ | JKOM | **Worse:** neither KOA nor pain: 36.2±13.9, KOA only :44.2±20.4, KOA and pain:61.4±23.0 p<0.05 |
| Lam, 2000^69^ | COOP/WONCA | **Worse:**  **Physical fitness:**  **Control group:** 76 (40%), **KOA:** 152 (71%) p<0.001.  **Feelings:**  **Control group:** 35 (18%), **KOA:** 50 (23%)  **Daily activity:**  **Control group:** 27 (14%), **KOA:** 50 (23%) p<0.05.  **Social activity:**  **Control group:** 17 (9%), **KOA**: 28 (13%).  **Overall health:**  **Control group:** 33 (17%), **KOA:** 53 (25%). |
